# Formation of Dislocations and Stacking Faults in Embedded Individual Grains during In Situ Tensile Loading of an Austenitic Stainless Steel

**DOI:** 10.3390/ma14205919

**Published:** 2021-10-09

**Authors:** Benjamin Neding, Darren C. Pagan, Johan Hektor, Peter Hedström

**Affiliations:** 1 Department of Materials Science and Engineering, KTH Royal Institute of Technology, 11428 Stockholm, Sweden; pheds@kth.se; 2 Cornell High Energy Synchrotron Source (CHESS), Ithaca, NY 14853, USA; dcp99@cornell.edu; 3 Deutsches Elektronen-Synchrotron (DESY), 22607 Hamburg, Germany; johan.hektor@mau.se

**Keywords:** high-energy X-ray diffraction microscopy, XRD line profile analysis, in situ deformation, metastable austenitic steels, stacking faults

## Abstract

The formation of stacking faults and dislocations in individual austenite (fcc) grains embedded in a polycrystalline bulk Fe-18Cr-10.5Ni (wt.%) steel was investigated by non-destructive high-energy diffraction microscopy (HEDM) and line profile analysis. The broadening and position of intensity, diffracted from individual grains, were followed during in situ tensile loading up to 0.09 strain. Furthermore, the predominant deformation mechanism of the individual grains as a function of grain orientation was investigated, and the formation of stacking faults was quantified. Grains oriented with [100] along the tensile axis form dislocations at low strains, whilst at higher strains, the formation of stacking faults becomes the dominant deformation mechanism. In contrast, grains oriented with [111] along the tensile axis deform mainly through the formation and slip of dislocations at all strain states. However, the present study also reveals that grain orientation is not sufficient to predict the deformation characteristics of single grains in polycrystalline bulk materials. This is witnessed specifically within one grain oriented with [111] along the tensile axis that deforms through the generation of stacking faults. The reason for this behavior is due to other grain-specific parameters, such as size and local neighborhood.

## 1. Introduction

Austenitic stainless steels are of considerable engineering importance due to their excellent corrosion resistance and mechanical properties [1,2]. In these steels, it is known that the stacking fault energy ($\gamma_{SF}$) can be used to predict the predominantly active deformation mechanism controlling the material’s mechanical properties. During plastic deformation, austenitic stainless steels with a low $\gamma_{SF}$ are known to form large stacking faults and undergo deformation-induced martensitic phase transformation responsible for the well-known transformation-induced plasticity (TRIP). Two martensitic products can form where the ε-martensite (hcp) is mainly of importance for promoting the formation of α′-martensite (bcc), which in turn provides significant strain hardening and contributes mainly to the TRIP effect [3,4,5]. Since ε-martensite is associated with the periodic ordering of stacking faults on every second fcc {111} lattice plane, stacking faults play a key role in the formation of ε-martensite and thus in the deformation of austenitic stainless steel. Therefore, enhanced knowledge of the formation and evolution of stacking faults and their relation to the deformation-induced martensitic transformations is crucial to understand the deformation behavior of not only austenitic stainless steel, but also other steels containing the austenite phase, and in particular when the TRIP or the twinning-induced plasticity (TWIP) effect is exploited. The chemical composition of the austenite and the deformation temperature have a significant effect on the $\gamma_{SF}$ [2,6,7]. However, it is known that stacking faults, ε-martensite and α′-martensite, in polycrystalline materials do not form homogenously in the bulk and instead grain size, grain morphology, grain orientation and grain neighborhood can play a role in their formation. Thus, the predominantly active deformation mechanism varies across the bulk [8,9,10] and it is necessary to consider all the mentioned parameters to predict the active deformation mechanism on the local scale. Despite its importance, the understanding of the deformation of individual grains within polycrystalline samples with respect to grain orientation and grain neighborhood is still vague. The incomplete understanding is partially due to the under-utilization of experimental methodologies capable of resolving the 3D microstructure non-destructively during in situ deformation. When using dominant conventional techniques such as scanning electron microscopy (SEM) or transmission electron microscopy (TEM), it is only possible to probe the surface (SEM) or thin foils (TEM) in situ. These regions do not reflect the bulk response, and additionally, there may be pronounced stress and strain relaxation effects [11]. A powerful method capable of resolving individual grains and sub-grains in the bulk of polycrystalline materials is high-energy X-ray diffraction microscopy (HEDM). It provides the possibility to follow, for example, the microstructure evolution non-destructively during deformation under consideration of grain position, orientation and neighborhood of individual grains within the bulk. Therefore, it captures the true-bulk response to plastic deformation [12]. The application of grain-resolved far-field HEDM (ff-HEDM) to study the deformation of austenitic stainless steel was presented by Hedström et al. In that work, the transformation of individual austenite grains into ε-martensite [10] and α′-martensite with respect to grain-resolved strain and grain orientation was investigated [13]. The combination of HEDM and peak-shape analysis opens up further opportunities to study the evolution of defects, such as dislocations and stacking faults in individual grains under consideration of grain orientation, neighborhood and morphology, as well as bulk-specific properties such as chemical composition and temperature. Pantleon et al. [14] demonstrated the power of this approach in their study on a polycrystalline Al specimen subjected to tensile strain. 

In the present work, we apply the combination of ff-HEDM and line profile analysis during in situ tensile loading to study the orientation dependence of the formation and evolution of stacking faults and dislocations in individual grains embedded in austenitic stainless steel. 

## 2. Materials and Methods

The composition of the investigated metastable austenitic stainless steel determined prior to deformation is given in Table 1. The analysis was performed by X-ray spectrometry (Cr, Ni), optical emission spectrometry (Mo, Ti) and combustion (C, N). The steel was supplied as hot-rolled strips. These strips were cut, cold-rolled and thereafter annealed at 1050 °C for 10 min to achieve a grain size of approximately 40 µm prior to further investigations. The fully austenitic steel was prepared as a dog-bone-shaped specimen with a gauge length of 3 mm and a gauge width of approximately 1 mm by electrical discharge machining. The tensile specimen was then ground and polished to a thickness of approximately 0.8 mm. 

The ff-HEDM experiment during uniaxial tensile loading was performed at the Cornell High Energy Synchrotron Source (CHESS) Ithaca, NY, USA, at the FAST (ID-3A) beamline. The experiment was conducted with a monochromatic X-ray beam energy of 61.332 keV. The illuminated gauge section of the sample was limited by rectangular slits with a size of 0.15 × 2 mm (H × W), resulting in a probed volume of 1 mm × 0.8 mm × 0.15 mm. The diffraction patterns were collected with two Dexela 2923 area detectors (3888 × 3072 pixels, 74.8 × 74.8 µm^2^ per pixel, and a sample-to-detector distance of approximately 1000 mm). Interrupted loading was performed in displacement control with a strain rate of 5 × 10^−4^ s^−1^ using the Rotation and Axial Motion System (RAMS2) load frame [15]. During pauses in loading, 2D diffraction patterns were collected in rotation increments of 0.25° while continuously rotating the sample 360° around the vertical axis by angle ω, resulting in 1440 frames per load step. The applied stress was measured by a load cell, and the strain was determined by digital image correlation. The analysis and reconstruction of the diffraction data were performed with the aid of the HEXRD software [16]. A detailed description of the HEDM reconstruction procedure can be found in [16,17].

**Figure 1:**
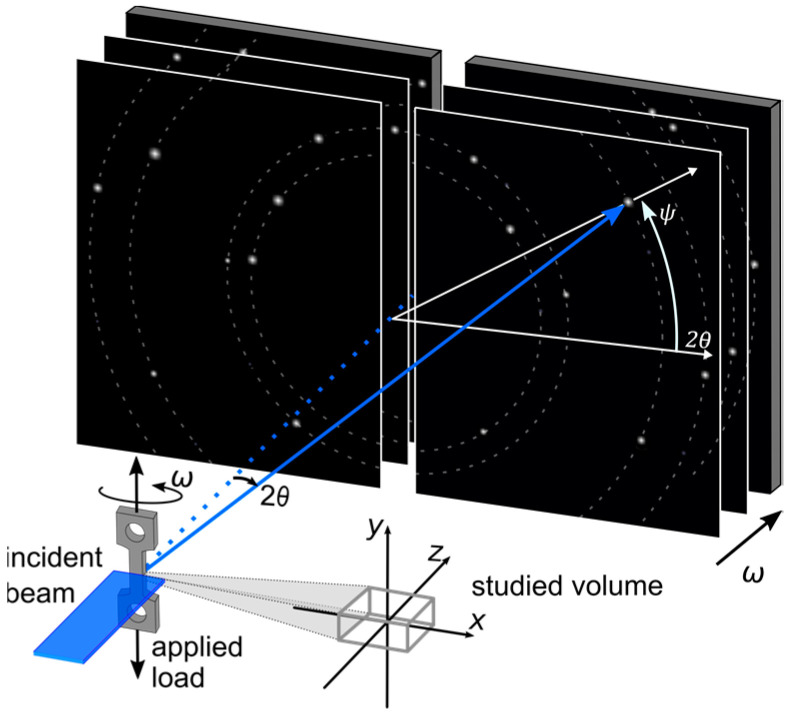
Experimental set-up for ff-HEDM.

During an ff-HEDM experiment, a multitude of component reflections (*hkl*) for families of lattice planes {hkl} are recorded. After the ff-data reconstruction, the (*hkl*) of a grain of interest was fitted. This was carried out within the HEXRD software. Each (*hkl*) was first integrated along ω (see Figure 1). To avoid peak overlap, the diffraction intensity of the (*hkl*) of interest was integrated over three successive detector images at $\omega_{i-0.25}$, $\omega_{i}$ and $\omega_{i+0.25}$, where the highest diffraction intensity of the (*hkl*) was observed at $\omega_{i}$. Secondly, the summed 2-dimensional intensities were integrated along the azimuthal direction on the detector, ψ, in order to generate 1-dimensional peaks. Thereafter, a pseudo-Voigt function was fitted to each reflection individually in 2θ space, from which the peak position and integral breadth of each component reflection at the specific strain can be determined. The integral breadth is determined by the area under a diffraction peak divided by the peak height.

The formation of stacking faults and dislocations was studied by plotting the integral breadth of each (*hkl*) versus the magnitude of its diffraction vector, g. Simm [18] showed, with the formalism derived by Balogh et al. [19], that the diffraction peak broadening introduced by the presence of stacking faults or dislocations depends on the {hkl} in a particular manner. This is based on the fact that the displacement field around a dislocation is anisotropic. Thus, the broadening of a diffraction peak introduced by the dislocation’s displacement field is given by the type of dislocation described by Burger’s vector, b, as well as its relation to g, the slip normal **n** and the dislocation line **s**. In a powder diffraction pattern, this effect is expressed by the average contrast factor ${\bar{C}}_{hkl}$, given as

(1) $${\bar{C}}_{hkl}=C_{h00}\left(1-qH^{2}\right),$$

where $C_{h00}$ and q depend on the elastic constants and the type of dislocation present and $H^{2}=\left(h^{2}k^{2}+h^{2}l^{2}+k^{2}l^{2}\right)/{\left(h^{2}+k^{2}+l^{2}\right)}^{2}$. 

**Figure 2:**
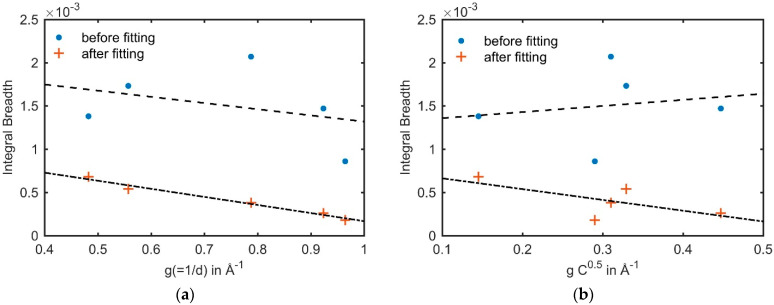
Example of the fitting procedure for grain #29 at 0.09 nominal strain. (**a**) Conventional WH plot and (**b**) modified WH plot.

It follows that a perfect dislocation in fcc crystals with Burger’s vector and slip plane [110]{111} affects the broadening of the {200} and {311} significantly more compared to the {220}, whereas the effect on the broadening of the {111} and {222} is only minor. This introduces an “M-shape” in the integral breadth versus the magnitude of g plot for {111}, {200}, {220}, {311} and {222}. This shape was observed and connected to dislocations many times in the literature [18,20,21,22]. In the case of stacking faults, the broadening of diffraction peaks is additionally affected by a size effect [23], i.e., broadening due to very small, coherent crystallite sizes, determined by the constant $\omega_{hkl}^{\;}$. Considering intrinsic and extrinsic stacking faults, the broadening of the {220} is affected almost twice as much as the broadening of the {111} and {222} and approximately 0.15 times as much as the {200} and {311} [18]. Thus, the integral breadth versus g plot determined from a deformed fcc material with the presence of stacking faults results in a distinct “hook-shape”. However, not all (*hkl*) are affected by the presence of stacking faults or dislocations to the same extent [23,24] since the lattice distortion responsible for an observable peak broadening is orientation-dependent with respect to g, thus inducing different peak-broadening for different (*hkl*). In order to have a better representation and comparison of the results, the median of the integral breadth of all (*hkl*) was calculated. With the median, the average density of planar faults in a single grain can be quantified utilizing the modified Williamson–Hall (WH) plot [18,20,25], since the extent of the strain anisotropy and thus the apparent broadening anisotropy is directly related to their density. The quantification of α furthermore underpins the qualitative view from the shape of the integral breadth versus g plot. The modified WH plot takes both ${\bar{C}}_{hkl}$ and $\omega_{hkl}^{\;}$ into account and, moreover, includes the fitting parameter $\beta^{\prime }=\frac{1.5\alpha -\beta }{a}$, where α is the stacking fault probability, β the twinning fault probability and a is the lattice parameter. The planar fault density can be determined by adjusting β′ for each grain (at a considered nominal macroscopic strain) in order to achieve the best linear fit during the modified WH plot fitting procedure. The fitting procedure was furthermore compared and verified by a conventional WH plot, in which the line broadening is plotted versus g, and the anisotropy is corrected by $\omega_{hkl}^{\;}$ and β′ only, i.e., the anisotropy due to dislocations is not included. An example of the fitting is given in Figure 2, where the median of the integral breadth of grain #29 at 0.09 nominal strain before the fitting procedure is represented by solid blue circles, and after fitting in orange crosses, utilizing the conventional WH plot (Figure 2a) and the modified WH plot (Figure 2b). The dashed lines represent the linear regressions of the integral breadth. It can be seen, that for both the conventional as well as the modified WH plot, the linear regression achieves a better fit after including planar faults in the fitting procedure.

## 3. Results and Discussion

**Figure 3:**
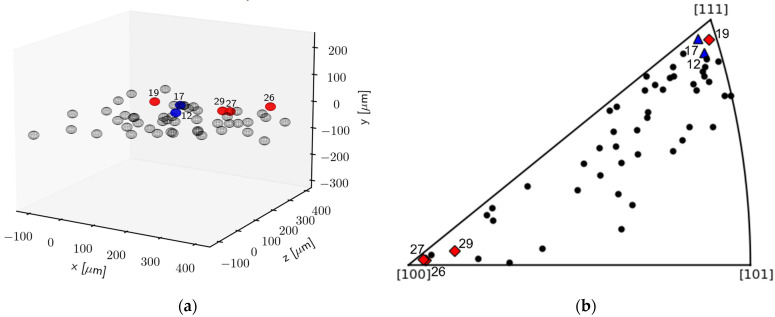
(**a**) Center of mass at 0.09 strain and (**b**) grain orientations in an inverse pole figure plot at 0.09 nominal strain. Red markers indicate grains which formed predominantly stacking faults, and blue markers represent grains in which the formation of dislocations was predominant.

Figure 3a shows the center of mass positions of the grains reconstructed from the ff-HEDM experiment. It shows the position of the studied grains within the studied volume at 0.09 strain. The grain orientations relative to the loading direction at 0.09 strain are given in Figure 3b. The red square marker indicates individual grains in which predominantly stacking faults were observed, whereas the blue triangle indicates grains with predominantly dislocations.

**Figure 4:**
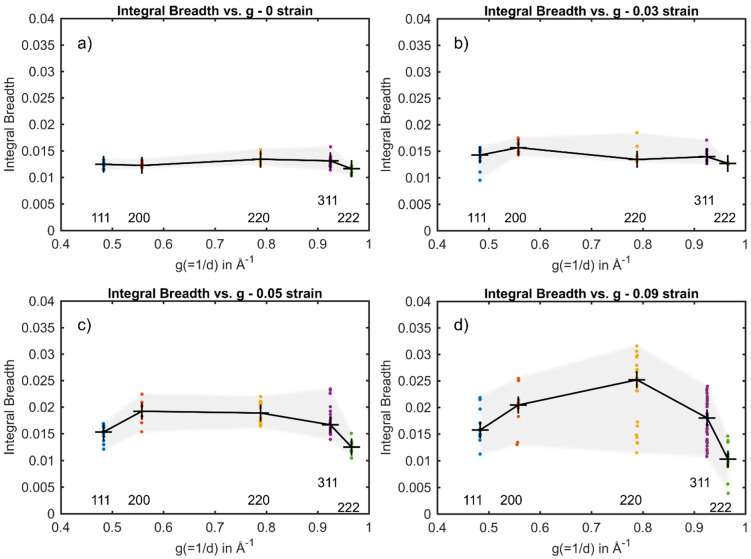
Integral breadth as a function of g for grain #29 at (**a**) 0 nominal strain, (**b**) 0.03 nominal strain, (**c**) 0.05 nominal strain and (**d**) 0.09 nominal strain.

**Figure 5:**
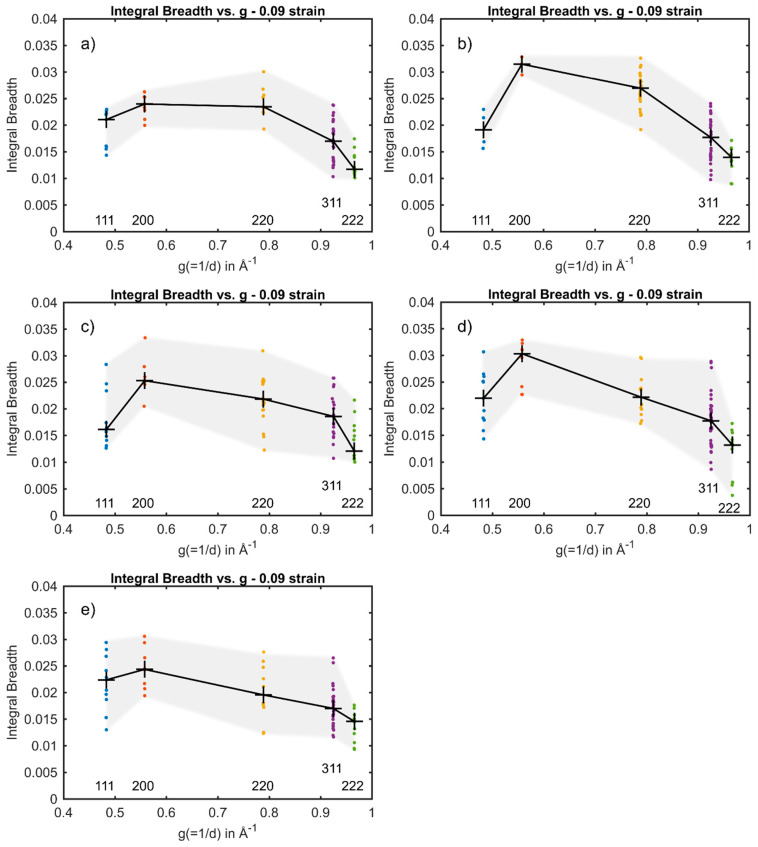
Integral breadth as a function of g at 0.09 nominal strain for (**a**) grain #19, (**b**) #26, (**c**) #27, (**d**) #12 and (**e**) #17.

The integral breadth of component reflections of {111}, {200}, {220}, {311} and {222} from individual embedded austenite grains in the steel plotted versus g are shown in Figure 4 and Figure 5. The median of the component reflection’s integral breadth is highlighted as a black cross. Figure 4a–d show the integral breadth versus g for grain #29 at 0–0.09 nominal strain, respectively. At 0 and 0.03 nominal strain (Figure 4a,b), the integral breadth of the component reflections from grain #29 has only a slight spread around the median. At 0 nominal strain, no planar fault could have been fitted to the plot, whereas at 0.03, α was determined to be α=0.01 × $10^{-3}$ (Table 2). This indicates that no significant amount of stacking faults formed at low strains. However, the broadening anisotropy in Figure 4b suggests the presence of predominantly dislocations since the strain anisotropy of dislocations introduces an “M-shape”, in which the integral breadth of the {200} and {220} is slightly larger compared to their neighboring reflections {111}, {220} and {222}. At 0.05 nominal strain, the integral breadth increases for all (*hkl*) compared to at 0.03 nominal strain (Figure 4c). However, the integral breadth determined for the component reflections of the {220} increases to a greater extent with increasing nominal strain, resulting in the transition from the “M-shaped” anisotropy to an anisotropic broadening that emerges due to the presence of stacking faults appearing as a “hook-shape”. This increase in the formation of stacking faults is also suggested by a slight increase in the determined α at 0.05 nominal strain to α=0.05 × $10^{-3}$. This shape transition of the integral breadth versus g plot was also found in a prior study by Neding et al. using average-grain high-energy X-ray diffraction measurements [26], where the formation of stacking faults was connected to the transition from “M shape” to “hook shape”. This transition becomes more obvious at 0.09 nominal strain, in which the median of the integral breadth of the {220} component reflections is significantly larger compared to {111}, {200}, {311} and {222}, leading to a distinct “hook-shape” (Figure 4d). In addition, the experimentally determined α increases considerably to α=1.17 × $10^{-3}$. This indicates that stacking faults are generated rapidly within the individual austenitic grain #29 between 0.05 and 0.09 nominal strain and dominates the plastic deformation. This transformation sequence has been observed before in austenite [27,28,29]. It is suggested that at low strains, the critical stress needed to generate partial dislocations and to ease their separation to form wide stacking faults is not reached, and thus the stacking faults are not recognized by XRD. At higher strains, stacking faults are generated, and dislocations dissociate into partial dislocations to form wide stacking faults. Thus, the density of faulted planes increases and their presence can be detected by XRD.

From Figure 3, it can be seen that austenite grains deformed along the [100] are found to form stacking faults, whereas in grains oriented with [111] along the tensile direction, the formation of stacking faults is impeded, and the formation of perfect dislocations is dominant. This was studied by investigating the anisotropic broadening of the diffraction peaks as a function of g at 0.09 nominal strain. Individual grains at 0.09 nominal strain oriented with [100] along the loading direction, #29, #19, #26 and #27 in Figure 4d and Figure 5a–c, respectively, show the same anisotropic broadening effect in the integral breadth plotted versus the magnitude of g leading to a “hook shape”. Besides the shape of the anisotropy, the experimentally determined α for grains #29, #19, #26 and #27 with values of 1.17 × $10^{-3}$, $0.86\times 10^{-3}$, 1.29 × $10^{-3}$ and $0.96\times 10^{-3}$, respectively, also suggests the presence of significant amounts of stacking faults. In contrast, the integral breadth of the component reflections of the {220} from grain #12 and grain #17 oriented with their [111] along the loading direction at 0.09 nominal strain (see Figure 5d,e) are significantly smaller as compared to {200} and approximately the same magnitude as {311}. Therefore, the plot implies the “M shape”, indicating the presence of predominantly dislocations. Furthermore, the magnitude α was determined to be considerably smaller. In grain #12 and in grain #17, α was determined to be α=0.26 × $10^{-3}$ and 0.24 × $10^{-3}$, respectively (see Table 2). However, stacking faults with an amount comparable to the grains oriented with their [100] parallel to the loading direction, were found in grain #19, even though grain #19 is oriented with its [111] along the loading direction. 

The observed orientation-dependent deformation behavior can be explained by the fact that the partial dislocations bounding a stacking fault, referred to as leading and trailing dislocation, experience different resolved shear stress [8,30,31].

Table 3 shows the Schmid factors for all possible partial dislocations with the direction of <$\bar{1}2\bar{1}$> on {111} calculated for tension in [111] and [001] using MTEX [32]. It can be seen that the difference in Schmid factor for the leading and trailing partial dislocation in grains oriented with their [001] along the loading direction is with 0.2357 larger compared to the difference in Schmid factor in grains oriented with their [111] along the loading direction, with 0.1571. Thus, the partial dislocations in grains oriented with their [001] along the loading direction separate readily and more faulting occurs. In contrast, the partials in grains with the [111] along the loading direction have a lower Schmid factor difference between the leading and trailing partial dislocation, which leads to a smaller separation distance between the partials. Thus, the formation of stacking faults in grains oriented with their [111] along the loading direction is less likely. Extensive faulting in grains with the <001> parallel to the external load was also observed by Goodchild et al. [8], who studied the transformation of grains in textured metastable austenite steels ex situ. However, as it can be seen in Figure 3, the formation of stacking faults occurs in grain #19, which is oriented with their [111] along the loading direction. This indicates that, even though the difference in the Schmid factor due to the grain’s orientation with respect to the loading direction indicates the formation of predominantly dislocations, the separation of partial dislocations; with that, the formation of stacking faults can still occur. The occurrence of a different deformation mechanism in grains with similar orientations might be due to differences in size and local grain neighborhood, which can affect the deformation behavior in addition to grain orientation. This observation emphasizes that it is crucial to consider the combination of all effects in order to reliably predict the deformation behavior of individual grains and therefore the deformation behavior of the bulk. 

## 4. Conclusions

Dislocations and stacking faults have been investigated in individual grains embedded within a polycrystalline bulk austenitic stainless steel. In situ X-ray line profile analysis was successfully applied to six individual grains with different orientations with respect to the external load. The integral breadth of more than 88 diffraction peaks per loading step was extracted for each individual grain from which the predominant deformation mechanism and stacking fault probability was deduced. It was shown that the orientation of individual grains has a significant impact on the predominant deformation mechanism for nominal strain up to 0.09. The formation of stacking faults was observed in grains oriented with their [100] along the loading direction, resulting in α=0.96 ×$\;10^{-3}$ − 1.29 × $10^{-3}$, whereas in grains oriented with their [111] along the loading direction, the presence of mere dislocations and only a small amount of stacking faults could be observed. This observation is believed to be related to the Schmid factor of partial dislocations. Orientations with a large difference in Schmid factor are more prone to form large stacking faults compared to grains with a small difference. Furthermore, the predominant deformation mechanism of an individual grain was followed as a function of external load. It was observed that at low nominal strain, plastic deformation occurs predominantly by dislocation, whereas with increasing nominal strain, the formation of stacking faults becomes prevailing, leading to α=1.17 × $10^{-3}$ at 0.09 nominal strain. It was furthermore revealed that the grain orientation alone is not sufficient to predict the deformation behavior and additional factors such as grain size and neighborhood must also be considered.

## Figures and Tables

**Table 1:** Chemical composition (in wt.%) of the investigated alloy.

Fe	Cr	Ni	C	N	Mo	W
bal.	17.9	10.4	0.004	0.033	0.02	0.02

**Table 2:** α determined utilizing the modified and regular WH plot in individual grains.

	Nom. Strain	Grain #29	Grain #19	Grain #26	Grain #27	Grain #12	Grain #17
α(×10^−3^)	0	N/A	-	-	-	-	-
	0.03	0.01	-	-	-	-	-
	0.05	0.05	-	-	-	-	-
	0.09	1.17	0.86	1.29	0.96	0.26	0.24

**Table 3:** Schmid factors of partial dislocations on the {111} with tension along [111] and [001].

u	v	w	h	k	l	σ||[111]	σ||[001]
−1	2	−1	1	1	1	0	−0.2357
−1	−1	2	1	1	1	0	0.4714
2	−1	−1	1	1	1	0	−0.2357
2	−1	1	1	1	−1	0.1571	0.2357
−1	−1	−2	1	1	−1	−0.3143	−0.4714
1	−2	−1	1	1	−1	−0.1571	−0.2357
1	2	−1	−1	1	1	0.1571	−0.2357
−2	−1	−1	−1	1	1	−0.3143	−0.2357
−1	1	−2	−1	1	1	−0.1571	−0.4714
−1	1	2	1	−1	1	0.1571	0.4714
−1	−2	−1	1	−1	1	−0.3143	−0.2357
−2	−1	1	1	−1	1	−0.1571	0.2357

## Data Availability

The data presented in this study are available on request from the corresponding author.
